# RES-Q-Trace: A Mobile CEAS-Based Demonstrator for Multi-Component Trace Gas Detection in the MIR

**DOI:** 10.3390/s18072058

**Published:** 2018-06-27

**Authors:** Norbert Lang, Uwe Macherius, Henrik Zimmermann, Sven Glitsch, Mathias Wiese, Jürgen Röpcke, Jean-Pierre H. van Helden

**Affiliations:** Leibniz Institute for Plasma Science and Technology (INP), Felix-Hausdorff-Str. 2, 17489 Greifswald, Germany; macherius@inp-greifswald.de (U.M.); hzimmermann@inp-greifswald.de (H.Z.); glitsch@inp-greifswald.de (S.G.); mathias.wiese@inp-greifswald.de (M.W.); roepcke@inp-greifswald.de (J.R.); jean-pierre.vanhelden@inp-greifswald.de (J.-P.H.v.H.)

**Keywords:** quantum cascade laser, interband cascade laser, absorption spectroscopy, cavity-enhanced spectroscopy, trace gas sensing

## Abstract

Sensitive trace gas detection plays an important role in current challenges occurring in areas such as industrial process control and environmental monitoring. In particular, for medical breath analysis and for the detection of illegal substances, e.g., drugs and explosives, a selective and sensitive detection of trace gases in real-time is required. We report on a compact and transportable multi-component system (RES-Q-Trace) for molecular trace gas detection based on cavity-enhanced techniques in the mid-infrared (MIR). The RES-Q-Trace system can operate four independent continuous wave quantum or interband cascade lasers each combined with an optical cavity. Twice the method of off-axis cavity-enhanced absorption spectroscopy (OA-CEAS) was used, twice the method of optical feedback cavity-enhanced absorption spectroscopy (OF-CEAS), respectively. Multi-functional software has been implemented (i) for the general system control; (ii) to drive the four different laser sources and (iii) to analyze the detector signals for concentration determination of several molecular species. For the validation of the versatility and the performance of the RES-Q-Trace instrument the species NO, N_2_O, CH_4_, C_2_H_4_ and C_3_H_6_O, with relevance in the fields of breath gas analysis and the detection of explosives have been monitored in the MIR with detection limits at atmospheric pressure in the ppb and ppt range.

## 1. Introduction

For more than a decade, a broad variety of applications invigorating efforts to further improve the sensitivity of technologies for trace gas detection have been required. This applies not only to the development, optimization and controlling of industrial production processes, but also to the field of medical breath analysis [1] and to the detection of illegal substances, such as drugs and explosives [2], where detection sensitivities down to part per billion (ppb) and part per trillion (ppt) levels are required. For example, the composition of exhaled human breath consists of major compounds such as N_2_, CO_2_, H_2_O, and O_2_ as well as volatile organic compounds (VOCs) at ppm levels or below. About four hundred VOCs occur only at ppb or ppt levels [1].

For the realization of portable, reliable, and selective sensor systems providing sufficient sensitivity for online and in situ trace gas detection, laser spectroscopic techniques are the method of choice. The usage of laser sources operating in the mid-infrared (MIR, 3–20 µm), such as quantum cascade lasers (QCLs) or interband cascade lasers (ICLs), enables access to the strong fundamental vibrational bands of many molecular species and, therefore, opens up the possibility of detecting them up to two orders of magnitude more sensitively because of the stronger absorption cross sections compared to the near-infrared (NIR). Hence, this has led to the establishment of mid-infrared laser absorption spectroscopy (MIR-LAS) as a highly effective diagnostic tool for sensitive trace gas detection. Sensors based on MIR-LAS have created a tremendous prospect in fundamental and applied studies allowing highly sensitive and selective spectroscopic measurements for species concentration determination. Enabled by the commercial readiness of QCLs and ICLs and their continuous wave (cw) operation at room temperature their application brought significant improvements in MIR-LAS, not only for trace gas detection [3,4,5], but also for studies in chemical physics [3], characterization of plasmas [6], breath gas analysis [7], and the investigations of supersonic expansions [8]. Equipped with specially designed distributed feedback (DFB) structures QCLs and ICLs operate in single-frequency mode providing continuous mode-hop free wavelength tuning. Nowadays, DFB-QCLs and ICLs are available with output powers up to hundreds of mW and laser linewidths down to hundreds of kHz. Although the laser frequency of these types of lasers can be designed over almost the entire MIR spectral range, the total spectral range covered by a single DFB-QCL or ICL is typically less than 7 cm^−1^ (see [3] and references therein). External cavity QCLs (EC-QCL) being QCLs without such a DFB structure but combined with an external grating provide wide tuning ranges of more than 100 cm^−1^ at high power levels and narrow linewidth [9]. Basically, EC-QCLs allow for multi-component detection. At each position of the grating controlled by a stepping motor a single spectrum covering about 1 cm^−1^ is obtained by tuning the grating with the help of a piezo actuator. However, the repeatability of such spectral scans is often limited by the inevitable hysteresis of the stepping motor. Therefore, to enable multi-component detection of trace gases in the MIR several lasers must be implemented in one sensor [10,11,12]. Recently, the successful design and application of so-called dual-wavelength QCLs was reported [13]. Operating in a time-division multiplexed mode, Jagerska and co-workers achieved a detection limit of 0.5 and 1.5 ppb for NO_2_ and NO, respectively, demonstrating a cost-effective and compact setup for the simultaneous detection of two trace gases.

A further important issue is the possibility of ensuring very low limit of detection (LOD) values at a high dynamic range combined with a sufficient accuracy. The application of cavity-enhanced spectroscopy (CES) techniques using dielectric mirrors to build a high finesse optical cavity is a powerful approach to realize a LOD at parts-per-billion (ppb) or parts-per-trillion (ppt) levels.

Following the demonstration of the achievement of very low detection limits with setups in the NIR based on optical path lengths in the order of kilometers [14], the successful combination of cavity-enhanced absorption techniques with cw ICLs, QCLs and external-cavity (EC) QCLs could be proven recently [15,16,17]. Miller and co-authors reported the detection of formaldehyde down to 150 ppb within 3 s acquisition time using off-axis cavity-enhanced absorption spectroscopy (OA-CEAS, being a special variant of CEAS, see also Section 2.1) with an ICL [15]. Van Helden and co-workers demonstrated a LOD for CH_4_ of 6 ppb at an effective optical path length of 1.78 km in a simple CEAS sensor using a cw EC-QCL as MIR radiation source [17].

This detection limit has been essentially improved up to the ppt range applying optical feedback cavity-enhanced absorption spectroscopy (OF-CEAS) [18,19,20,21,22]. In OF-CEAS, the self-locking of a laser to an optical cavity is used not only to increase the optical path length but also to enhance the cavity transmitted power [23,24].

To exploit the capabilities of OA-CEAS and of OF-CEAS in the MIR spectral range for highly sensitive, effective, and reliable trace gas detection, a compact and transportable sensor system (RES-Q-Trace) was developed. The concept of the RES-Q-Trace system is to have a versatile and mobile multi-component trace gas detection system. In a modular approach, two in principle identical optical platforms were designed comprising one OA-CEAS and one OF-CEAS measurement cell each. Each measurement cell can be equipped either with a QCL or with an ICL resulting in four different lasers in total. Therefore, at least 4 species can be detected simultaneously with this system. The design allows the lasers and the mirrors of the cavities to be changed according to the measurement task. All measurement cells sample from the same volume in parallel allowing for multi-component detection. Multi-functional software has been implemented not only for the general system control, but also to drive the four different laser sources and to analyze the detector signals for the determination of the gas concentrations.

In this paper, a description of the optical unit, the controlling and data processing as well as the analysis of the data is presented. The flexibility and versatility of RES-Q-Trace is demonstrated with examples of species density measurements with relevance for breath gas analysis and for the detection of explosives: NO, CH_4_, C_2_H_4_, C_3_H_6_O and N_2_O. The particular relevance of the selected species together with their required LOD is listed in Table 1.

The accuracy and limitations of the system are discussed based on Allen-Werle analysis of the noise in case of zero absorption in the cavity.

## 2. Materials and Methods

### 2.1. CES Techniques

Cavity-enhanced techniques using high-finesse optical cavities play an important role in spectroscopy when driven by sensitivity issues, the interaction time of light and matter, and therefore the interaction length must be improved significantly. By applying CES techniques such as OA-CEAS, OF-CEAS or noise-immune cavity-enhanced optical heterodyne molecular spectroscopy (NICE-OHMS), very low detection limits can be achieved. An extensive overview about the principles and applications of CES techniques including further reference in literature can be found in two recent scientific books [11,34].

#### 2.1.1. OA-CEAS

To improve the sensitivity achievable with CEAS, Paul and co-authors described a variant of this technique, where the laser beam is directed at an angle with respect to the cavity axis [35]. This so-called off-axis CEAS (OA-CEAS) or alternatively, off-axis integrated-cavity-output spectroscopy (OA-ICOS) aims to meet a re-entrant condition of the laser beam in the cavity by using a well-defined optical geometry. Consequently, the spectral density of the cavity modes increases because of an effective decreased cavity-mode spacing. Thus, the noise due to averaging the cavity frequencies is reduced in the resulting absorption spectra. As ordinary CEAS, OA-CEAS benefits from the increase in effective optical path length without locking to a single longitudinal cavity mode. Typical values of achievable minimum detectable absorption coefficients, *α*_min_, are in the range of 10^−8^–10^−10^ cm^−1^ [36,37]. The limitation in sensitivity of OA-CEAS is given by the significantly reduced cavity transmitted power. This becomes critical when low-power lasers and room-temperature detectors must be used in the experimental cavity setup. To overcome this drawback Centeno et al. proposed an improved three mirror OA-CEAS configuration where the reflected light from the first cavity mirror is re-injected into the cavity with the help of a third mirror leading to an enhancement of the transmitted cavity power [38]. It could be demonstrated that such a scheme provides a 10 times increase in signal-to-noise ratio as compared to standard OA-CEAS.

#### 2.1.2. OF-CEAS

Another variant to enhance significantly the achievable sensitivity is the technique of OF-CEAS. In OF-CEAS, the self-locking of a laser to an optical cavity is used not only to increase the optical path length but also to enhance the cavity transmitted power [23,24]. The built-up of intensity in a cavity caused by interaction of the laser frequency with a cavity resonance leads naturally to a leaking back of radiation to the laser itself.

Under appropriate conditions, such an optical feedback can significantly reduce the emission line width of the laser, e.g., from MHz down to below 1 kHz, thus increasing the coupling efficiency which enhances the cavity throughput. In principle, the optical feedback acts as an injection seeding of the laser as along as its field is in-phase with the emitted field by the laser. This leads to a self-locking of the emitted laser frequency to a cavity resonance and to a narrowing of the laser linewidth. Typically, the laser remains locked to this frequency for a time that is much longer than the cavity ring-down time, which is of the order of several µs. If the current applied to the laser is ramped, the laser frequency locks to successive cavity modes if the feedback rate, defined as the ratio of feedback power to laser output power, is sufficient. As a result, the intensity pattern transmitted by the cavity is characterized by an envelope of consecutive cavity modes. Because their separation on frequency axis corresponds to the free spectral range (FSR) of the cavity, OF-CEAS spectra are calibrated per se for relative frequency. Due to the nearly transparent character of the resonant cavity with time very low values for *α*_min_ in the range of 10^−9^–10^−11^ cm^−1^ can be achieved [14,24].

### 2.2. Optical Unit

**Figure 1:**
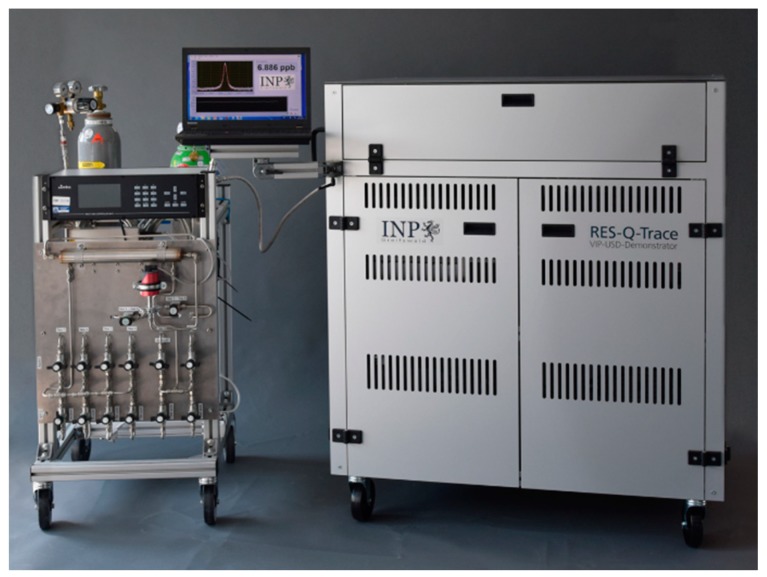
Mobile trace gas detection system RES-Q-Trace with gas mixing module.

A photo of the mobile demonstrator system RES-Q-Trace with a gas-mixing module used for test measurements is presented in Figure 1.

**Figure 2:**
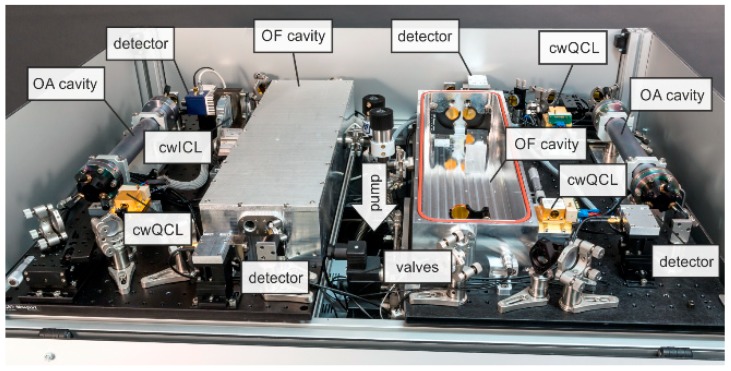
Detailed view at the optical unit of the RES-Q-Trace instrument comprising of two mirrored optical platforms. Details of one optical platform are shown in Figure 3.

**Figure 3:**
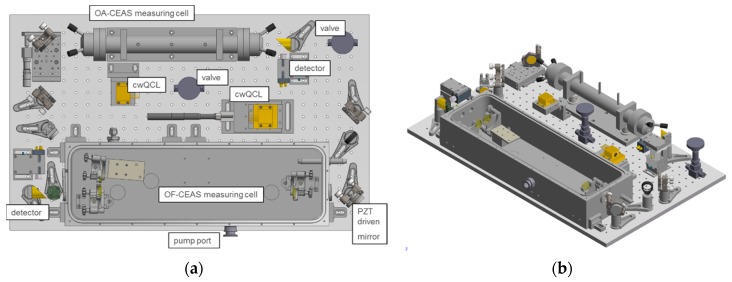
Modular platform comprising one OA-CEAS and one OF-CEAS measurement cell: (**a**) top view; (**b**) side view. The dimensions of one platform is 75 × 45 × 13 cm^3^.

The modular design of the optical unit implemented in the RES-Q-Trace instrument is given in Figure 2. The dimensions of the optical unit are 113 × 79 × 13 cm^3^ comprising two mirrored optical platforms equipped with one OA-CEAS and one OF-CEAS measurement cell each. A detailed view of one such optical platform is given in Figure 3a,b exemplarily.

All cavity setups are designed to be in a symmetric non-confocal geometry, i.e., the condition for optical stability, being 0<L<r or r<L<2r, where *L* is the distance between the mirrors and *r* the radius of curvature, is always fulfilled. Further details are given for example in [14].

The narrowband infrared emission of the four laser sources, either QCLs in industrial standard high heat load (HHL) packages (Alpes Lasers, St. Blaise, Swiss and AdTech Optics, City of Industry, CA, USA) or ICLs housed in a TO3 can (nanoplus, Gerbrunn, Germany) providing collimated infrared beams, is used to monitor the infrared absorption features of the target species. The temperature control of the thermoelectrically cooled lasers mounted on a custom made water-cooled heat sink is performed by an analog controller (Wavelength Electronics, Bozeman, MT, USA, PTC10K-CH) for each laser within milli-Kelvin precision in the range between −15 and +40 °C. Low-noise drivers with long-term reliability from Wavelength Electronics supply the current for the lasers (QCL1000 and QCL500) depending on their operation range. For the power supply of the current drivers, home-built analog devices are used to reduce electrical noise.

In the next sections the principle setup of both types of CEAS measurement cells, the common driver and supply unit as well as the data processing and analysis technique are described. For selected configurations depending on the target species, detailed results will be given in Section 3.

#### 2.2.1. OA-CEAS Measurement Cells

Both optical platforms comprise a setup with an OA-CEAS measurement cell, where the light from the laser source is coupled by two plane steering mirrors directly into the optical resonator cell. For the off-axis alignment, the last mirror is mounted additionally on a translational stage. Both OA-CEAS cavities consist of two high-reflective mirrors in special mirror mounts (CRD Optics) at a distance of 32 cm in an evacuable cell resulting in a free spectral range (FSR) of 468.4 MHz. The cavity mirrors have a specified reflectivity of better than 0.9995 over the whole working range of the laser with a 25.4 mm diameter and a radius of curvature of 1 m (CRD Optics, Lompoc, CA, USA and LohnStar Optics, Escondido, CA, USA). An off-axis parabolic mirror behind the cavity focuses the transmitted light onto a thermo-electrically cooled DC-coupled photovoltaic detector (VIGO, Ozarow Mazowiecki, Poland, PVI-4TE). The detector signal is additionally enhanced with a 100 MHz wideband voltage amplifier (Femto, Berlin, Germany, DHPVA-100) with a variable gain before being recorded by a fast 2 channel waveform digitizer card (Alazar Technologies, Pointe-Claire, QC, Canada, ATS330, 50 MS/s, 12 bit).

The lasers are scanned typically over 0.1–0.3 cm^−1^ by applying a sawtooth function at 1 kHz produced by a high speed arbitrary function generator board (Tabor Electronics, Irvine, CA, USA, 5300, 125 MS/s) for each laser. For a more efficient averaging of the cavity frequencies, a sinusoidal modulation frequency of 1 MHz is superimposed on the sawtooth function leading to an improved excitation of the cavity resonances and therefore into a smoother baseline. Both arbitrary function generator boards are synchronized via a trigger signal, which is also used to trigger the acquisition of the analog detector signal by the 2 channel waveform digitizer cards.

Relative frequency calibration can be provided by inserting temporarily a germanium etalon (FSR 1458 MHz) between the two steering mirrors before the cavity. The absolute frequency calibration is given via the measurement of a molecular absorption in the cavity with known line position.

#### 2.2.2. OF-CEAS Measurement Cells

For measurements where a higher sensitivity is required, an OF-CEAS measurement cell is implemented on each optical platform, see also Figure 3a,b respectively. The light from the laser is directed by two plane steering mirrors into the optical resonator cell with the last mirror being mounted on a piezoelectric transducer (PZT; Physik Instrumente, Karlsruhe, Germany, E-505.00) for fine control of the feedback phase. The two OF-CEAS measuring cells consist of sealed aluminum boxes with BaF_2_ windows, which were tilted with respect to the optical axis to reduce reflections returning to the laser. Inside the box, a V-shaped cavity with each cavity arm length being 40 cm is constructed from three high reflective mirrors. This corresponds to a FSR and therefore to a spectral sampling resolution of 187.5 MHz (0.00625 cm^−1^). The cavity mirrors have a specified reflectivity of better than 0.9995 over the whole working range of the laser with a 25.4 mm diameter and a radius of curvature of 1 m (CRD Optics, Lompoc, CA, USA and LohnStar Optics, Escondido, CA, USA). The laser-to-cavity distance is set equal to each cavity arm length to maintain the in-phase condition of the feedback field at the laser. Therefore, the laser source is mounted on a translational stage for a precise adjustment of the distance. The optical feedback rate to the laser is controlled with the help of an aperture stop in front of the steering mirror. The light leaving the OF-CEAS measuring cell is focused on a DC-coupled thermo-electrically cooled photovoltaic detector (neoplas control, Greifswald, Germany, IRDM-DCA).

The lasers are scanned typically over 0.3–0.53 cm^−1^ by applying a sawtooth function between 20 and 50 kHz provided by a data acquisition card (DAQ; National Instruments PCI-6221, 740 kS/s, 16 bit). The transmitted signals are recorded by the same DAQ card. A low-pass filter with a 3 dB frequency of 663 kHz is implemented before the input of the current driver to suppress additional digital noise caused by the DAQ card. In addition, a voltage divider between the DAQ card and the current driver provides the maximum digital resolution of the DAQ card to for driving the lasers.

**Figure 4:**
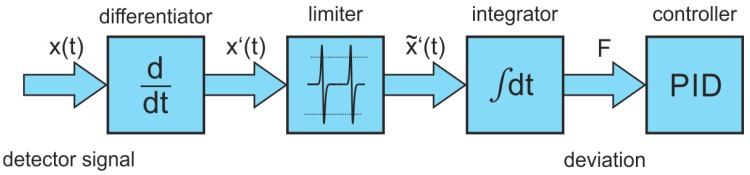
Scheme of the software routine used for controlling the optical feedback.

To maintain self-locking of the laser to the cavity resonances the optical feedback phase must be controlled actively. This is provided by controlling continuously the laser-to-cavity distance with the help of the above-mentioned PZT driven steering mirror in front of the cavity. As long as the ideal phase condition is not met, the transmitted signal of a cavity mode appears asymmetric because the laser locks to a frequency range, which is not centered on the peak of the cavity resonance. Therefore, an error signal is generated being proportional to the phase error, which is based on the median symmetry of the cavity modes in each recorded scan [18]. Using a LabVIEW (National Instruments, Austin, TX, USA) routine the detector signal is first differentiated point by point. Following a cutting of the signal at an experimentally determined level, the resulting values are integrated over the entire mode spectrum. This error signal is fed into a programmable PID control loop, which provides the flexibility to choose the P, I and D coefficients appropriately based on the experimental conditions. The output value of the PID control loop is scaled and converted to an analog voltage by a digital-to-analog converter on a slow multi-function DAQ board (National Instruments, Austin, TX, USA, PCI-6010) and fed into the PZT controller (Physik Instrumente, Karlsruhe, Germany, E-505.00) for maintaining phase-locking with a response time equal to the duration of a single scan. In Figure 4, the scheme of the software routine used for controlling the optical feedback is shown.

With proper aligned feedback rate, the relative frequency axis of the transmission spectra is given by the FSR of the cavity. Because of an efficient built up of power in the cavity due to the self-locking of the laser to the cavity, a mode matching of the laser beam to the cavity modes has been omitted as has been done often in past OF-CEAS studies [39,40].

#### 2.2.3. Common Driver and Supply Unit

In the RES-Q-Trace instrument, the two levels below the optical unit house central components for both modular optical platforms. This includes the gas management system with four electro-magnetic valves, two capacitive pressure sensors (Leybold, Köln, Germany, CTR 100 N) and a central pump (Edwards, Burgess Hill, UK, nXDS6iC). For the central sample gas inlet, two valves are implemented, one for setting the desired pressure coarsely (VACOM, Großlöbichau, Germany, 204.5VE5-14S-VV-S), while with the help of the second one (SMC, Egelsbach, Germany, VDW21-5G-3-01F-J-Q), installed behind the first one in a by-pass, the final pressure is set more precise but slower. In case of measuring sticky molecules, an extra inlet valve (VACOM, Großlöbichau, Germany, 204.5VE5-14S-VV-S) is implemented enabling separate purge cycles of all measurement cells with nitrogen. The removal of any gas sample occurs via a central outlet valve (MKS Instruments, München, Germany, CVNL-K1-ECLVV-24DC) between the pump and the measurement cells. The valves can be operated automatically with the help of a home-built controller providing programmed gas handling cycles for all measurement cells at once, e.g., the timing of gas inlet, measurement period, evacuating, purging with an inert gas, refilling, etc. A LAN interface enables the communication with the control computer and therefore the synchronization of the gas handling with the measurement.

The response time of the system is determined by the method with the longest acquisition time needed. As reported in Section 3, Table 2, this varied substantially among the species the system was validated for. For example, when detecting N_2_O in the gas sample with the highest achievable sensitivity, the measurement cycle of the total system is 500 s plus the time required for gas handling. If an additional purge cycle is needed, the measurement time lasts about 30 s longer.

All device drivers, i.e., for lasers, detectors, pressure sensors, and piezos including their power supplies as well as two water chillers (Solid State, Wappingers Falls, NY, USA, ThermoCube 300) and control computers are installed in the rack below the optical subsystem. A central power management (Raritan, Somerset, NJ, USA, Dominion PX8) provides turn-key operation as well as safety shut-downs. The total power consumption is about 2.7 kW.

**Figure 5:**
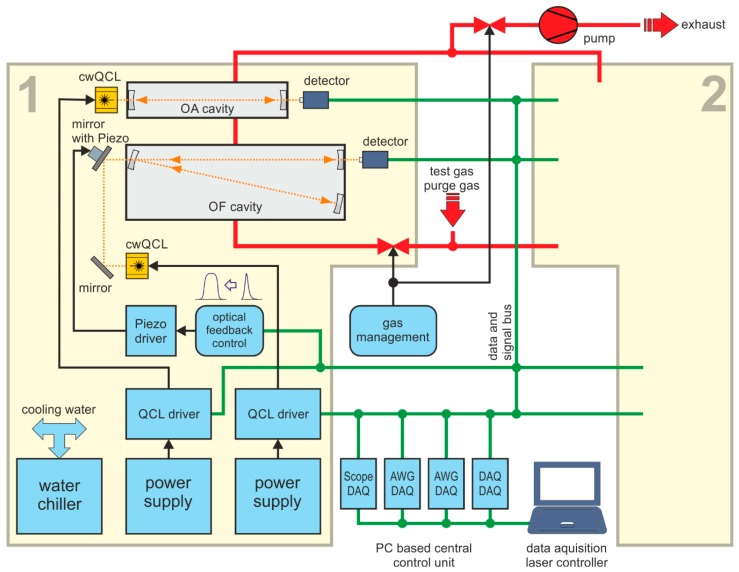
Scheme of the RES-Q-Trace system with OA- and OF-CEAS implementation, AWG—arbitrary waveform generator, DAQ—data acquisition board. The details are shown only for optical platform 1 (yellow box labeled with 1) as optical platform 2 (yellow box labeled with 2) is a mirrored setup of the first one.

In Figure 5, the scheme of the RES-Q-Trace system with OA- and OF-CEAS implementation is shown exemplarily for optical platform 1. Platform 2 is not shown in detail as it is a mirrored setup of platform 1. All measurement cells sample from the same volume in parallel allowing for multi-component detection. The RES-Q-Trace system features a stable wheel frame with four steering load rollers enabling a flexible operation site. The overall weight of the instruments amounts to about 470 kg.

### 2.3. Data Processing and Analysis Technique

The full experiment control as well as the acquisition, averaging and processing of data are performed using custom LabVIEW routines running on four integrated 1.66 GHz ATOM dual-core Mini-ITX computers (ADLINK Technology, Model MXC-4002D/M2G). They contain all required DAQ cards: one fast waveform digitizer (50 MS/s), one analog-to-digital board allowing continuous sampling with up to 740 kS/s, two high speed arbitrary function generator boards (125 MS/s) and four slow (200 kS/s) multi-function analog-to-digital boards. The access to the control computer unit takes place externally and user-defined via a LAN interface and a laptop.

The acquired data can be averaged according to the requirements. In case of OA-CEAS, a moving average is applied to integrate over chaotic mode structure, while maintaining the maximum throughput. From the measured transmitted intensity, the absorption coefficient *α* for OA-CEAS data is given as:

(1) $$\alpha \left(\nu \right)=\frac{I_{0}\left(\nu \right)-I\left(\nu \right)}{I\left(\nu \right)}\cdot \frac{\left(1-R\right)}{L}=\sigma \left(\nu \right)\cdot n$$

where *I*_0_ is the baseline signal, *I* is the transmitted signal with the sample present, *R* is the effective reflectivity of the cavity mirrors and *L* is the physical length of the cavity. With the knowledge of the frequency-dependent absorption cross section *σ*(*ν*) of the absorbing medium its concentration *n* can be determined.

In case of OF-CEAS, the evaluation of *α* follows a different relation assuming that sample absorption is small compared to mirror losses [18,23]:

(2) $$\alpha \left(\nu \right)=\left[\sqrt{\frac{I_{0}\left(\nu \right)}{I\left(\nu \right)}}-1\right]\cdot \frac{\left(1-R\right)}{L},$$

where *I* is the amplitude of each cavity mode and *I*_0_ the amplitude of the cavity modes representing the baseline, which has been determined by 2nd order polynomial fit of the amplitudes of both the first and last 8 modes of the scan.

In all experiments, the effective reflectivity *R* was determined separately by measuring the integrated absorption for several known amounts of a mixture of reference gases with documented line strengths [41,42]. For OF-CEAS spectra, this analysis had to be performed separately for even and odd modes to account for different phase at the folding mirror of the cavity. In the corresponding LabVIEW routine, a peak-finder algorithm is implemented allowing to detect and separate even and odd cavity modes in the acquired signal sequence. The absorption coefficients *α* derived independently for even and odd modes are then combined to the entire absorption spectrum.

The resulting spectra are displayed to the user and can be manually or automatically stored to the file system as well as analyzed online. For the analysis the spectra are fitted with a Voigt line profile for each transition using the Levenberg-Marquardt algorithm. In case of OA-CEAS spectra, the Gaussian width is set as the square root of the sum of the Doppler linewidth at room temperature and an unknown part caused by an additional purely Gaussian broadening due to the current modulation of the laser [17]. The Lorentzian part of the Voigt profile is a free running fit parameter to compensate for differences in the pressure broadening of the line width induced by nitrogen instead of air as is typically listed, for example in the HITRAN database [41]. In contrast, the fitting of OF-CEAS spectra is not influenced by any instrumental broadening. Because of the self-locking of the laser to a mode of the cavity with a width of a view kHz (depending on the ring-down time, see e.g., [14]), the laser linewidth is narrowed down to this range as described in Section 2.1.2. In addition, there is no need for an additional current modulation in OF-CEAS. Therefore, in fitting a Voigt profile to OF-CEAS spectra the Gaussian width is set to the Doppler broadening at room temperature only, whereas the Lorentzian part is treated as a free running fit parameter as in the case of OA-CEAS spectra. In case of spectrally unresolved transitions, a cumulative fit of all components is performed with fixed relative center frequencies and relative areas as well as Gaussian linewidths caused by Doppler broadening only. From the results for the area values of the profile and the known line strength the concentration of the sample is determined.

For the characterization of the ultimate sensitivity and the temporal stability of the individual CEAS setups, the minimum detectable absorption coefficient *α*_min_ was measured as a function of the acquisition time *τ* (number of scans/tuning rate). According to common practice, *α*_min_ was derived by analyzing the noise of the baseline, i.e., in case of zero absorption [19,43,44]. For example, Gorrotxategi-Carbajo and co-workers argue that using this method possible variations in non-zero concentrations of the interesting species can be avoided leading to an overestimation of the detection limit [19]. The cavity was filled with pure N_2_ at a pressure the concentration measurements were performed at. Analyzing the statistics of the noise, the LOD results from the standard deviation (1*σ*).

## 3. Results

For the validation of the performance of the RES-Q-Trace instrument five different molecular species, NO, N_2_O, CH_4_, C_2_H_4_ and C_3_H_6_O, have been monitored with detection limits in the ppb and ppt range. In Table 2, the studied trace gases, which play a role in medical breath analysis and for the detection of explosives, are given together with spectral position, applied CEAS method, laser type, minimum detectable absorption coefficient *α*_min_ and limit of detection at atmospheric pressure. The latter is derived from the relation $\alpha_{min}=\sigma_{peak}\cdot n_{min}$ with $\sigma_{peak}$ being the peak absorption cross section calculated from the line strength and the corresponding pressure-broadening coefficient, both given in [41].

In the following, the detection of N_2_O, CH_4_ and C_3_H_6_O is described exemplarily in detail as representatives of each CEAS method implemented in the RES-Q-Trace system. For OA-CEAS two examples will be discussed for the case of measuring an absorbing gas with a single spectrally resolved line profile and for a broadband absorber resulting in a spectrally unresolved congested spectrum.

### 3.1. OA-CEAS

#### 3.1.1. Spectrally Resolved Single Absorption Line Profile

**Figure 6:**
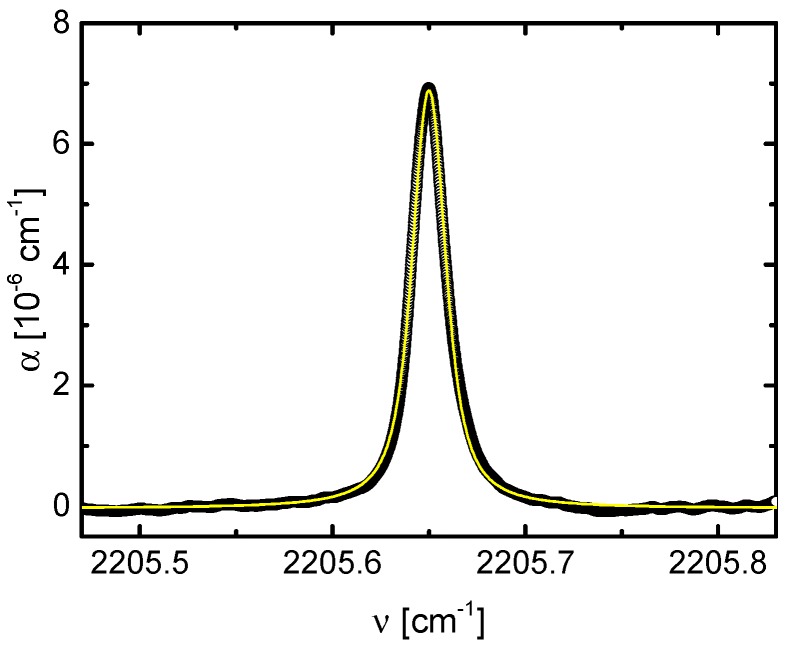
OA-CEAS spectrum of 200 ppb N_2_O diluted in 25 hPa N_2_ after 100,000 averages with a tuning rate of 200 Hz.

Figure 6 shows an OA-CEAS spectrum of a single N_2_O transition at 2205.65 cm^−1^ for 200 ppb N_2_O diluted in N_2_ and measured at a pressure of 25 hPa. The spectrum results from 10^5^ averages at a tuning rate of the QCL of 200 Hz. The effective reflectivity, *R*, of the mirrors was determined by measuring the integrated OA-CEAS signal $\int d\nu (I_{0}-I)/I$ for total pressures in the range from 10–200 hPa and plotted as a function of the known concentration of N_2_O. From the slope of the linear fit the value of *R* could be obtained using Equation (1). From these measurements, an effective mirror reflectivity of 0.99972 ± 0.00003 was determined, which corresponds to a finesse of the cavity of $\pi \sqrt{R}/\left(1-R\right)=11,218$ and an effective path length of 1143 ± 122 m.

**Figure 7:**
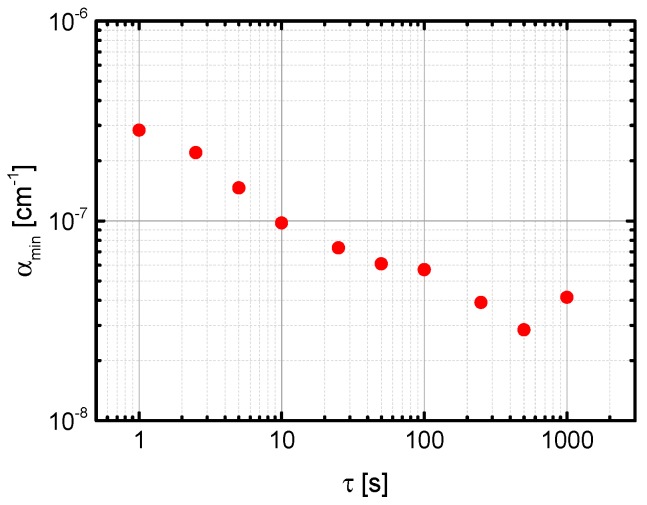
Allen-Werle deviation plot of the minimum detectable sensitivity *α*_min_ for N_2_O depending on the acquisition time. For *τ* being 500 s, *α*_min_ equals to 2.8 × 10^−8^ cm^−1^ which corresponds to a LOD at *p* = 1013 hPa of 7 ppb Hz^−1/2^.

The result of the analysis of the LOD as described in Section 2.3 is shown in Figure 7. The best value of *α*_min_ = 2.8 × 10^−8^ cm^−1^ was obtained for 100,000 averages recorded in 500 s. Related to atmospheric pressure this value corresponds to a LOD of about 0.3 ppb.

#### 3.1.2. Spectrally Unresolved Congested Absorption Spectrum

**Figure 8:**
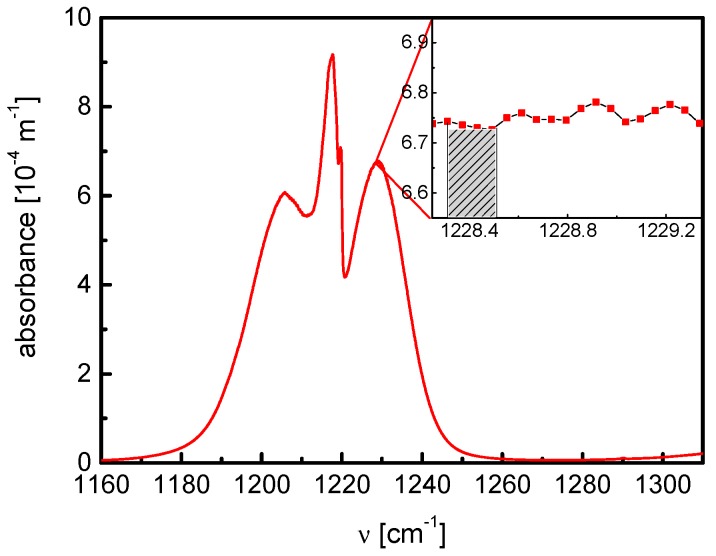
Congested FTIR spectrum of 1 ppm·m^−1^ C_3_H_6_O at 1220 cm^−1^ [42]. Inset: spectral range used for sensing of C_3_H_6_O via OA-CEAS with a cw-QCL; effective absorption cross section: *σ* = 4.7 × 10^−20^ cm^2^.

The capability to detect higher molecular species characterized by complex congested absorption spectra is demonstrated using acetone as an absorber in an OA-CEAS module of the RES-Q-Trace system. In Figure 8, a complex FTIR spectrum of 1 ppm·m^−1^ C_3_H_6_O from the Pacific Northwest National Laboratory (PNNL) IR database in the spectral range 1160 to 1260 cm^−1^ is presented [42]. The hatched area in the inset shows the spectral range from 1228.335–1228.508 cm^−1^ used for monitoring of C_3_H_6_O via OA-CEAS with a cw-QCL. From the PNNL data an effective absorption cross section of *σ* = 4.7 × 10^−20^ cm^2^ could be derived for this spectral range.

The reflectivity of the mirrors in the cavity was determined using a mixture of 5 ppm C_3_H_6_O diluted in N_2_ measuring the absorbance at different total pressures. According to Equation (1), from the linear regression the mean effective reflectivity was found to be 0.99938 ± 0.00001 resulting in a finesse of 5066 and an effective path length of about 518 m in this cavity. With the analysis of the CH_4_ transition at 1228.793 cm^−1^ measured in a sample of ambient air in the same pressure range as acetone, the reliability of these results could be confirmed assuming the concentration of CH_4_ in air in Greifswald is 1.8 ppm [17].

**Figure 9:**
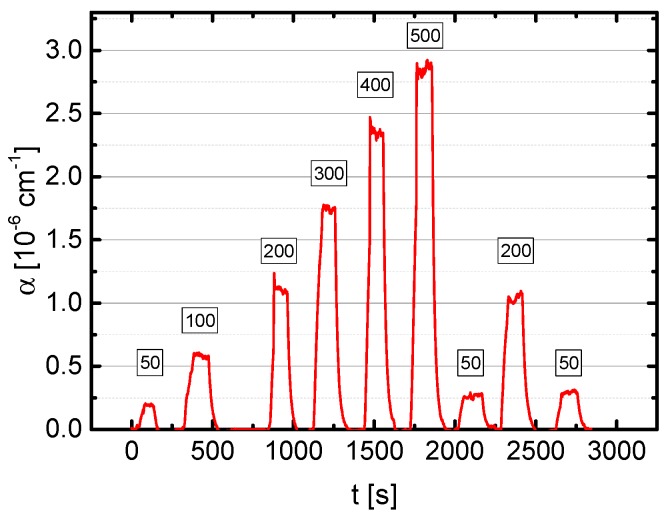
Absorption coefficient of 5 ppm C_3_H_6_O at 1228.4 cm^−1^ diluted in N_2_ measured at different total pressures in a range of 50 to 500 hPa; minimal detectable absorption: *α*_min_ = 3 × 10^−8^ cm^−1^, LOD at 1013.25 hPa: 10 ppb·Hz^−1/2^.

The resulting absorption coefficient of 5 ppm C_3_H_6_O diluted in N_2_ at 1228.4 cm^−1^ measured at different total pressures and at a tuning rate of the cw-QCL of 1000 Hz is shown in Figure 9. The minimal detectable absorption coefficient was found to be *α*_min_ = 3 × 10^−8^ cm^−1^. This value corresponds to a LOD of 10 pbb Hz^−1/2^ at atmospheric pressure.

### 3.2. OF-CEAS

**Figure 10:**
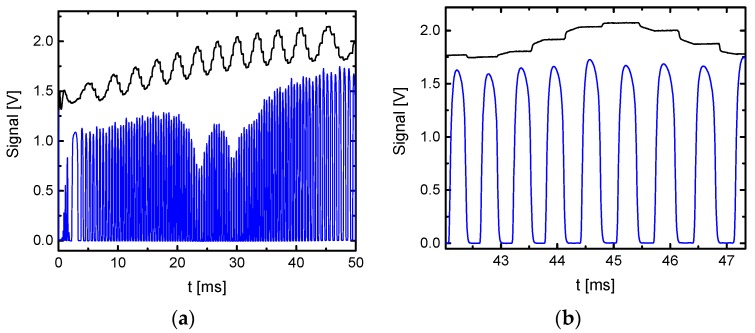
Left panel (**a**)—cavity transmission for 300 ppb CH_4_ in N_2_ as buffer gas at 1353 cm^−1^ at a total pressure of 300 hPa while the laser is scanning to lower wavenumber. This transmission spectrum is the average of 1000 scans. Right panel (**b**)—a magnification of the modes around 45 ms. The locking time of the modes was ∼430 µs and the time between modes was ∼220 μs. In both panels, the upper trace is an etalon trace showing flat sections when locking to the modes occurs (shifted for clarity) [22].

Finally, the validation of the performance of the OF-CEAS modules is demonstrated by high sensitive detection of CH_4_. In Figure 10, a typical cavity transmission spectrum of CH_4_ at 1353 cm^−1^ averaged over 1000 scans of a sample at a pressure of 300 hPa with 300 ppb CH_4_ in N_2_, used as buffer gas, is presented [22]. For about 45 ms, the cw-QCL locked to about 100 successive cavity modes with a locking time of about 430 μs, which is much longer than the ring-down time of the cavity being typically below 10 μs. The time between single modes was in the range of 220 μs, see also the right panel in Figure 10.

Furthermore, amplitude oscillations of the transmitted signal for alternating even and odd modes having different phases at the folding mirror, and therefore slightly different reflectivity can be clearly seen. The upper traces in both panels of Figure 10 represent the simultaneous recorded transmission of a germanium etalon in a by-pass. As long as the laser locks to one of the modes of the cavity due to optical feedback, the transmitted signal remains constant with time.

**Figure 11:**
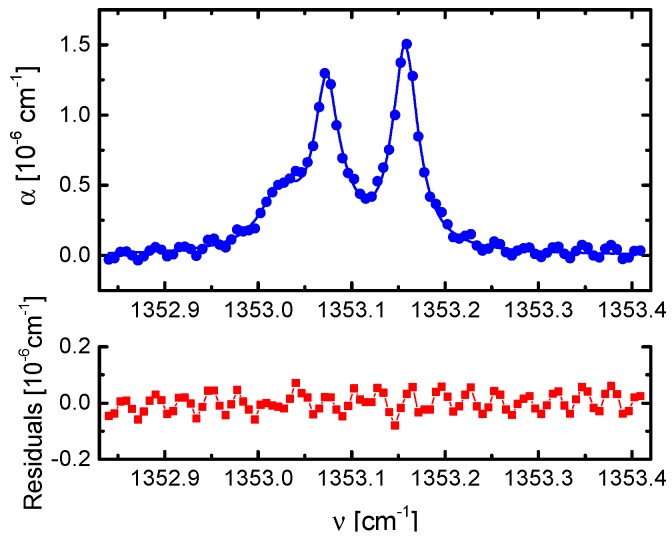
Upper panel: Absorption coefficient spectrum consisting of both even and odd modes of 300 ppb of CH_4_ in N_2_ as a buffer gas at a total pressure of 300 hPa together with a composite fit considering the 4 transitions of CH_4_ which contribute to the observed absorption feature. Lower panel: the residual of the fitted absorption profile; the standard deviation of the residual of this fit gives *α*_min_ = 3.35 × 10^−8^ cm^−1^ [22].

For the determination of the effective mirror reflectivity, the absorption of a 500 ppb sample of CH_4_ diluted in N_2_ was measured at different total pressures. According to the method described above, a value of 0.9997 was found for even modes and 0.99971 for odd modes, respectively. The resulting mean value of 0.99971 corresponds to an optical cavity finesse of 5416 and to an effective path length of 2760 m [22]. Figure 11 shows a cumulative fit to the measured absorption coefficient spectrum of 300 ppb CH_4_ in N_2_ as buffer gas at a total pressure 300 hPa averaged over 1000 scans, together with the residual of the fitted absorption profile.

**Figure 12:**
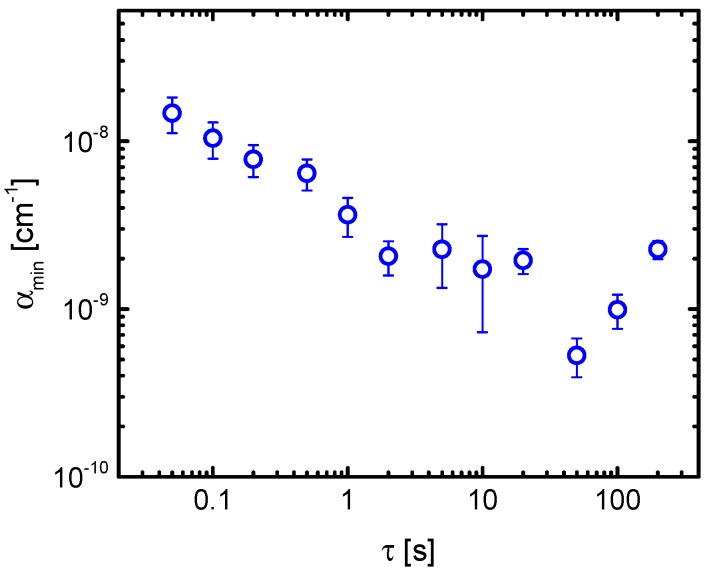
Allan-Werle deviation plot of the minimum detectable sensitivity, *α*_min_, derived from the comparison of a stored background to the cavity transmission for each mode, as a function of the acquisition time *τ*. The cavity was filled with pure nitrogen at a pressure of 300 hPa. The smallest value of *α*_min_ corresponds to a LOD of 39 ppt at atmospheric pressure (adapted from [22]).

The sensitivity as well as the temporal stability of the OF-CEAS setup was characterized according to the method described in Section 2.3. Because of a distorted baseline by a sinusoidal structure (being visible in Figure 11), a background spectrum was measured and stored for the same number of scans and used for the calculation of *α* according to Equation (2) [22]. Such sinusoidal structured baselines are often observed in CEAS and CRDS when optical interference with parasitic reflections in the setup could not be minimized, e.g., [45,46]. In Figure 12, the mean value for *α*_min_ is plotted against the acquisition time *τ* whereas its standard deviation serves as an uncertainty for this value. The lowest value of *α*_min_ of (5.3 ± 0.1) × 10^−10^ cm^−1^ was obtained for 1000 averages recorded in 50 s. This corresponds to a minimum detectable concentration of 39 ppt of CH_4_ at atmospheric pressure [22].

## 4. Discussion

In the past, several approaches were reported to combine three or four quantum cascade lasers to an instrument with multi-component detection capabilities [10,47,48,49]. To enhance the sensitivity multipass optical cells were used, partially in combination with techniques such as wavelength modulation spectroscopy [48]. For the discrimination of the laser beams passing through the multipass optical cell time, multiplexing techniques must be applied, although this could be realized in the kHz regime.

The main goal of this work was to proof and validate the concept using quantum cascade lasers as well as interband cascade lasers in combination with optical cavities for a mobile, reliable, and selective sensor system, which is sensitive for the online detection of multiple trace gases down to the ppt level. The RES-Q-Trace system developed for the simultaneous detection of at least four molecular species was tested for NO, N_2_O, CH_4_, C_2_H_4_, and C_3_H_6_O. The targeted species are important not only in the field of monitoring pollutant emissions, but also in medical breath analysis and for the detection of explosives. In a modular approach, two optical platforms were designed comprising one OA-CEAS and one OF-CEAS measurement cell each. All measurement cells sample from the same volume in parallel allowing for multi-component detection. The design allows the lasers and the mirrors of the cavities to be changed according to the measurement task. Hence, other species such as NO_2_, OCS, HCHO, and O_3_ could be targeted, whereby the performances of the system must be validated for these cases.

The achieved sensitivities (listed in Table 2) are in the area of application of most of these substances for the investigations of respiratory diseases. In particular, methane and acetone are known as indicators for intestinal diseases and diabetes respectively, see also Table 1. Therefore, in breath gas analysis a LOD of 20 ppb for methane is required, whereas in case of acetone 25 ppb are sufficient. Acetone of that amount is considered also as sufficient for its stand-off detection from objects or persons as it is an essential precursor for the production of triacetone triperoxide (TATP). Furthermore, nitrous oxide and nitric oxide play important roles as decay products in the detection of nitrogen-based explosives, such as trinitrotoluene (TNT). It could be shown, that the required LOD in the range of several hundred ppt could be achieved at least for the detection of N_2_O. In case of ethane, the targeted sensitivity of 10 ppt required for its measurement in human breath as an indicator of oxidative stress could clearly not be achieved. The main obstacle to get to higher sensitivities using an optical cavity was caused by significant losses due to increasing absorption and scattering in the ZnSe substrate of the high-reflective mirrors at wavenumbers of 1000 cm^−1^.

For wavenumbers greater than 1100 cm^−1^, such losses are no longer dominant and less sensitive on the spectral range. For example, the achieved value of *α*_min_ of (5.3 ± 0.1) × 10^−10^ cm^−1^ in 50 s for the detection of CH_4_ at 1353.1 cm^−1^ corresponding to a LOD of 39 ppt at atmospheric pressure is the smallest value reported for the detection of CH_4_ using OF-CEAS.

Interband cascade lasers emitting at wavenumbers above 2500 cm^−1^ are attractive because they enable access to the strong C-H and O-H stretch vibrational modes, which opens the possibility of detecting very sensitive chemical compounds down to the ppt range like aromatics, alkanes, alkenes, alcohols, phenols, and water to name a few. Quantum cascade lasers are commercially not available in this spectral region. As a proof of principle, the first application of an ICL used in an OF-CEAS measurement for the detection of methane was shown as a part of this work [21]. Successful improvements of this approach were reported recently in applying ICLs in OF-CEAS for the measurement of SO_2_ and NO respectively [50,51].

## Figures and Tables

**Table 1:** Studied trace gases with their relevance in breath gas analysis and for detection of explosives, and the required LOD for them.

Gas	Relevance	Required LOD [ppb]	Reference
NO	lung and respiratory diseases	0.5	[25]
	decomposition product of nitrogen-based explosives, such as TNT, AN, PETN, and RDX ^1^ to name a view	0.001–1	[26,27]
N_2_O	decomposition product of nitrogen-based explosives, such as TNT, AN, PETN, and RDX ^1^ to name a view	0.001–1	[26,27]
CH_4_	intestinal diseases	20	[28,29]
C_2_H_4_	oxidative stress	0.01	[30]
C_3_H_6_O	diabetes	25	[31]
	precursor of TATP ^2^	25	[32,33]

^1^ TNT—Trinitrotoluene; AN—Ammonium nitrate; PETN—Pentaerythritol tetranitrate; RDX—Trimethylenetrinitramine; ^2^ TATP—Triacetone triperoxide.

**Table 2:** Studied trace gases with vibrational mode number, transition, spectral position, CEAS method, laser type, minimum detectable absorption coefficient, its measurement time and corresponding limit of detection (LOD) at atmospheric pressure.

Gas	*v*	Transition	Spectral Position [cm^−1^]	CEAS Method	Laser	*α*_min_ [cm^−1^]	Measurement time [s]	LOD @ 1013 hPa [ppb]
NO	1	R(5.5) doublet	1897.0	OA-CEAS	QCL	3.5 × 10^−8^	1	2
N_2_O	4	P(20)	2205.7	OA-CEAS	QCL	2.8 × 10^−8^	500	0.3
CH_4_	4	11 F_1_ 9 ← 12 F_2_ 1	1230.1	OA-CEAS	QCL	1.3 × 10^−8^	1	22
	3	7 A_1_ 8 ← 6 A_2_ 1 7 F_1_ 27 ← 6 F_2_ 1 7 E 8 ← 6 E 1	3086.1	OF-CEAS	ICL	7.9 × 10^−7^	2	3
	4	9 A_2_ 1 ← 8 A_1_ 1 9 F_2_ 2 ← 8 F_1_ 1 9 E 1 ← 8 E 2	1353.1	OF-CEAS	QCL	5.3 × 10^−10^	50	0.04
C_2_H_4_	7	Q (18)	986.0	OA-CEAS	QCL	3.9 × 10^−8^	1	39
C_3_H_6_O	17	C-C stretch unresolved	1228.3–1228.5	OA-CEAS	QCL	3.0 × 10^−8^	1	10
